# Setting Boundaries for Statistical Mechanics

**DOI:** 10.3390/molecules27228017

**Published:** 2022-11-18

**Authors:** Bob Eisenberg

**Affiliations:** 1 Department of Applied Mathematics, Illinois Institute of Technology, Chicago, IL 60616, USA; bob.eisenberg@gmail.com; 2 Department of Physiology and Biophysics, Rush University Medical Center, Chicago, IL 60612, USA

**Keywords:** statistical mechanics, Maxwell equations, variational methods, EnVarA, boundary conditions

## Abstract

Statistical mechanics has grown without bounds in space. Statistical mechanics of noninteracting point particles in an unbounded perfect gas is widely used to describe liquids like concentrated salt solutions of life and electrochemical technology, including batteries. Liquids are filled with interacting molecules. A perfect gas is a poor model of a liquid. Statistical mechanics without spatial bounds is impossible as well as imperfect, if molecules interact as charged particles, as nearly all atoms do. The behavior of charged particles is not defined until boundary structures and values are defined because charges are governed by Maxwell’s partial differential equations. Partial differential equations require boundary structures and conditions. Boundary conditions cannot be defined uniquely ‘at infinity’ because the limiting process that defines ‘infinity’ includes such a wide variety of structures and behaviors, from elongated ellipses to circles, from light waves that never decay, to dipolar fields that decay steeply, to Coulomb fields that hardly decay at all. Boundaries and boundary conditions needed to describe matter are not prominent in classical statistical mechanics. Statistical mechanics of bounded systems is described in the EnVarA system of variational mechanics developed by Chun Liu, more than anyone else. EnVarA treatment does not yet include Maxwell equations.

## 1. Introduction

This paper is an expanded and reworked version of the preprint arXiv:211212550. 2021 [1].

Molecular systems nearly always involve electrical properties, because matter is held together by electrical forces, as specified by quantum chemistry. The role of electrical forces is obvious from the Schrödinger wave equation of the electron, that specifies quantum chemistry. The Schrödinger equation includes the electrical potential *V*. The Hellmann Feynman theorem makes this role of electricity more explicit as the source of forces in atoms and molecules [2,3]. The electricity of the Hellmann Feynman theorem and quantum mechanics is the electricity of the Maxwell equations, as [4] makes clear in all three volumes. The Bohm formulation of quantum mechanics makes this role particularly clear [5,6,7,8] as it is used in the design of actual devices [9,10,11,12]. The potential energy V of the Schrödinger equation (and quantum mechanics) probably should describe the energy of the entire electrodynamic field—magnetic and electrical because electrons have magnetic as well electrostatic properties, a permanent magnetic dipole (‘spin’) as well as charge. The potential energy should probably be specified by the Maxwell equations, dynamic as well as static—although the potential energy V is often computed using only Coulombic (i.e., electrostatic) energies.

Even uncharged atoms like argon interact through the quantum fluctuations of their charge density, which are stochastic deviations from the mean charge density of zero. These London dispersion forces are electrical. They are important determinants of macroscopic forces [13]. For example, quantum fluctuations in charge density in atom #1 induce polarization charge in a neighboring atom #2 that interact to produce macroscopic forces between the two atoms.

The Bohm formulation of quantum mechanics illustrates the role of electrodynamics in chemical and physical systems [5,6,8] in a practical way, less mysterious than in other forms of quantum mechanics. The Bohm formulation has proven useful and accurate enough to help design high speed transistors and semiconductor devices, reviewed and described in detail [14] for FETs (field effect transistors) which are the active elements of almost all modern digital devices. The unavoidable conclusion then is that theories, calculations, or simulations of molecules must satisfy the laws of electrodynamics. These laws are reviewed in [6,7,8,9,10,15,16,17] with notable issues dealt with in [5,18,19,20,21,22,23,24] among others.

The question then is what are the laws of electrodynamics that molecular simulations and statistical mechanics must satisfy?

**Why isn’t electrostatics good enough?** Molecular and atomic simulations use Coulomb’s law to describe electrical forces.

Coulomb’s law is a simple algebraic law that does not include time. It is a static description of electrodynamics and as such obviously cannot describe the dynamics of charges and the dynamic time dependent fields they produce, like the magnetic field.

(1) $$\mathrm{Electrical}\;\mathrm{Force}=\frac{1}{4\pi \varepsilon_{0}}\frac{q_{1}q_{2}}{r^{2}}$$

where the charges $q_{1}$ and $q_{2}$ at distance r produce the electrical force with electrical constant $\varepsilon_{0},$ the permittivity of free space. Coulomb’s law is more or less equivalent to the first two Maxwell equations Equations (2) and (3) (provided boundary conditions and realistic dielectric properties are included in both the law and the equations). However, the other two Maxwell equations Equations (4) and (5), involve time and Coulomb’s law does not. These Maxwell equations Equations (4) and (5) will almost always predict observables that are different at different times. However, Coulomb’s can give only one result at those different times. Thus, Coulomb’s law cannot describe the properties of electric fields that involve the Maxwell equations Equation (4) and the Maxwell Ampere law. 

The Maxwell-Ampere law [4,24] is of particular importance for several reasons

1. The magnetic field is inextricably coupled to the electric field by the theory of (special) relativity, as Einstein put it (p. 57, [25]) “The special theory of relativity… was simply a systematic development of the electrodynamics of Clerk Maxwell and Lorentz.” The Feynman lectures [4]—e.g., Section 13-6 of the electrodynamics volume 2 of [4]—and many other texts of electrodynamics and/or special relativity [26] elaborate on Einstein’s statement.
2. The Maxwell-Ampere law allows electrical phenomena to couple with magnetic phenomena to produce radiation (like light) that propagates through a vacuum containing zero matter and zero charge.
3. The Maxwell-Ampere implies that the divergence of the right hand side of Equation (5) is zero. The divergence of the curl is zero for any field that satisfies the Maxwell equations, as is proven in the first pages of textbooks on vector calculus. The reader uncomfortable with vector calculus can simply substitute the definitions of curl and divergence [27,28] into the relevant Equation (11) below and note the cancellation that occurs.
4. A field with zero divergence is by definition a field that is perfectly conserved, that can neither accumulate nor dissipate nor disappear. Thus, the right side of the Maxwell-Ampere law is a perfectly conserved quantity, an incompressible fluid whose flow might be called ‘total current’ [29,30]. Because the right hand side of the Maxwell Ampere law always includes a term $\varepsilon_{0}\partial E/\partial t$ that is present everywhere, even where charge and its flux J are zero, this term can provide the coupling needed to create radiation. The derivation of the radiation law (e.g., Equation (13) below) can be found in most textbooks of electrodynamics.
5. The conservation of total current is of great practical importance [31,32] because it can be computed in situations involving large numbers (e.g., 10^19^) of charges, where computation of Equation (2) is impossible because of the extraordinary number of charges and interactions (that are ***not*** just pairwise, see [33] and references therein). The continuity equation so important in fluid mechanics is thus more or less useless in studying the electrodynamics of material (and chemical) systems on an atomic scale.

**Magnetism does not have to be important.** The Maxwell-Ampere law is of great importance in purely electrostatic systems where magnetic forces are negligible. The conservation of total current is exact. It does not depend on the existence of substantial magnetic forces. The derivation of conservation from the Maxwell Ampere law is succinct and pleasing (see Equation (11) and preceding discussion) and the derivation is exact not depending on any statement of the size of the B field. And, of course, other derivations are possible.

The conservation of total current (i.e., the right hand side of the Maxwell Ampere law) is of the greatest importance throughout electrical engineering, in the form of Kirchhoff’s law, as is apparent from its presence as a keystone in the design of even the highest speed circuits of our computers [34,35,36,37,38,39,40] with general discussion in [31], where references are given to devices that function on the quantum scale. Kirchhoff’s law is best understood as a necessary consequence of the Maxwell equations [30], whether or not magnetism is important in a particular system.

**Figure 1:**
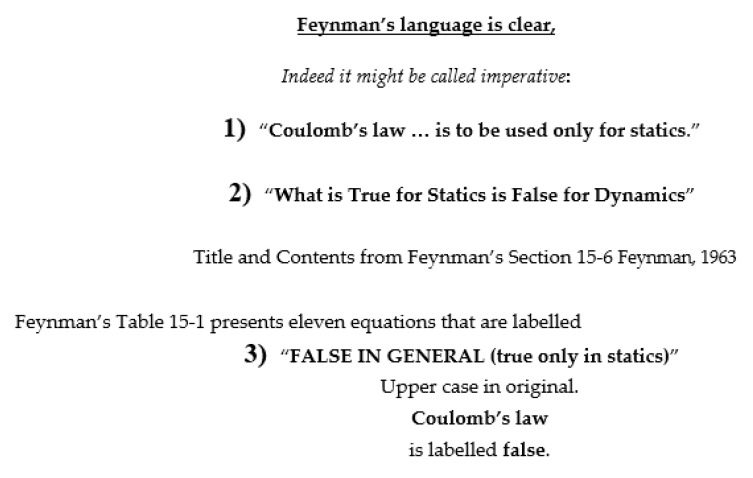
The need for electrodynamics, not just electrostatics, is emphasized by Feynman, in language that could hardly be more explicit. See volume 2 of [4].

**Limitations of Coulomb’s law.** The limitations of Coulomb’s law are not widely understood and so I find it necessary to give detailed citations to volume 2 of [4]. Figure 1 shows the most important quotations (and the exact references) documenting the limitations of Coulomb’s law.

Given the consequences of these facts, it is wise to pay attention to the imperative language of Feynman. His language borders on unprofessional, but the motivation seems obvious, in my opinion. Feynman is impatient with misuse of Coulomb’s law in time dependent systems and the large literature that ignores his Table 15–1.

Sadly, I must conclude that electrostatics cannot provide a sound foundation for statistical mechanics. Statistical mechanics involve atoms. Atoms move quickly [41]. (See the elegant discussion of the speed of sound, p. 845–853). Atoms are charges and so charge moves rapidly on the atomic scale of Ångstroms and femtoseconds. Electrostatics obviously is not enough to describe femtosecond events. Electrodynamics is needed.

## 2. Theory

**The laws of electrodynamics are the Maxwell equations**. The Maxwell equations (as written by Heaviside [42,43,44,45,46] and others [47,48,49,50]) are universal laws valid over an enormous range of times and distances. They are used to compute electrical forces within atoms and between stars. They form part of the Schrödinger equation of quantum mechanics [4] and are particularly prominent in its Bohm formulation [5,7,8,9,17,51,52]. The electrodynamic component of the quantum force of the Schrödinger and Bohm formulations is the same as the electrodynamic force computed between stars by the Maxwell equations, and probably the same as the electrodynamic force between galaxies.

**Core Maxwell Equations:**

(2) $$\mathrm{Gauss}\;\mathrm{Law}\;\;\;\;\;\;\;\;div\;E=\frac{\rho }{\varepsilon_{0}}$$

(3) Magnetic Monopoles=0   div B=0 

(4) $$\mathrm{Maxwell}\;\mathrm{Faraday}\;\mathrm{Law}\;\;\;curl\;E=-\frac{\partial B}{\partial t}$$

(5) $$\mathrm{Maxwell}\;\mathrm{Ampere}\;\mathrm{Law}\;\;curl\;B=\mu_{0}\left(J+\varepsilon_{0}\frac{\partial E}{\partial t}\right)$$

**Figure 2:**
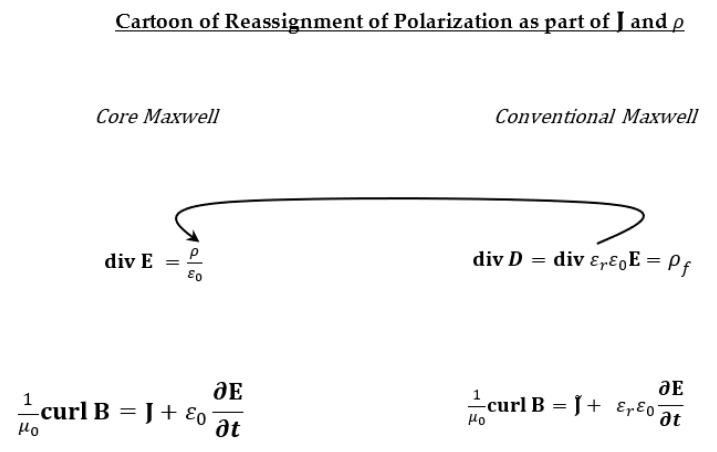
Classical and Core Maxwell equations. $\tilde{J}$ describes the flux of mass with charge, after the usual dielectric term is subtracted from J. $\rho_{f}$ describes the distribution of charge after the usual dielectric term is subtracted from ρ. The charge ρ describe all charges, however small, and all flux J (of charges with mass), however fast, brief, and transient. They include polarization phenomena in the properties of ρ and J whereas the classical equations use an oversimplified representation (see text) that describes the polarization of an idealized dielectric by its dielectric constant $\varepsilon_{r}$ with the appropriately modified definitions of charge and flux, namely the free charge $\rho_{f}\;\mathrm{and}\;\tilde{J}$.

The core Maxwell Equations (1)–(4) use the variables ρ and J to describe all charges, however small, and all flux (of charges with mass), however fast, brief, and transient. Equations (1)–(4) are called the core Maxwell equations because they include polarization phenomena in the properties of ρ and J rather than in the conventional way, shown in Figure 2. Structuring the Maxwell equations in that form as ‘Core Maxwell Equations’ makes clear that the equations are universal and not constitutive. Classical formulations of the Maxwell equations are constitutive equations that depend on the property of matter. The core Maxwell equations do not, except in the sense that they require a separate theory of polarization (and a separate theory of other effects of electrical and magnetic forces on the distribution of charge) just as the Navier–Stokes equations for compressible fluids require a separate theory for the effect of pressure on density [53,54,55,56].

Nonlinear terms are not present in the Maxwell equations. My understanding is that nonlinearity has not yet been observed in experiments, but is predicted at very large field strengths, approaching the Schwinger limit of some 1.32 × 10^6^ volts/Ångstrom.

**Classical Maxwell equations are constitutive equations.** The Classical Maxwell Equations embed the dielectric constant of matter $\varepsilon_{r}$ into the very definition of their variables. For example, the Maxwell vector field D is defined to include the polarization P of matter and the relative dielectric constant $\varepsilon_{r}$, a single dimensionless positive real number, sometimes called the relative permittivity.

(6) $$\;\;D≜\varepsilon_{0}E+P=\varepsilon_{0}\left(\varepsilon_{r}-1\right)E$$

(7) $$D≜\varepsilon_{0}E+P=\chi E=\varepsilon E\;$$

In these equations, ε is the (dimensional) permittivity. The electric susceptibility is $\chi =\varepsilon_{r}-1$.

**Physical meaning of *χ* and *ε*_0_ could not be more different.** The susceptibility χ is a property of matter. The permittivity of space $\varepsilon_{0}$ is a property of space, whether a vacuum or filled with matter.

The permittivity of space $\varepsilon_{0}$ is best viewed as a consequence of the theory of relativity (see any text on special relativity or [4]). It is truly a constant that does not vary under any known circumstances or conditions. It is called the electrical constant for that reason. The dielectric constant $\varepsilon_{r}$ is, on the other hand, a mixture containing a constitutive variable very sensitive to the properties of polarization in a particular material in a particular circumstance [57,58,59,60,61,62,63,64,65,66,67,68,69,70,71,72,73,74,75,76,77,78,79,80,81,82,83,84,85,86,87,88,89,90,91,92,93,94,95,96,97,98,99,100,101,102,103,104,105,106,107,108]. In my opinion, lumping parameters with such different physics (relativity vs. material science) in one variable is likely to produce confusion about the meaning of the lumped variable, as indeed has arisen in my experience.

The actual measured polarization properties of liquids can almost never be reasonably approximated by a dielectric constant $\varepsilon_{r}$ as documented in an extensive literature [29,57,58,59,60,61,62,63,64,65,66,67,68,69,70,71,72,73,74,75,76,77,78,79,80,81,82,83,84,85,86,87,88,89,90,91,92,93,94,95,96,97,98,99,100,101,102,103,104,105,106,107,108]. The literature of impedance spectroscopy is reviewed recently with extensive citations of the literature in [108]. That literature shows that the experimentally measured dielectric constant varies with the type of salt, the composition and concentration of mixtures of salt (as in the solutions in which life occurs), in complex ways that cannot be summarized easily as shown by the hundreds of measurements in some twenty volumes of data reported by Barthel, for example [62,63,109]. The literature showing enormous variation in polarization and thus in ‘effective dielectric constant’ extends far beyond the impedance spectroscopy of liquids and has for a very long time [57,58,59,60,61,62,63,64,65,66,67,68,69,70,71,72,73,74,75,76,77,78,79,80,81,82,83,84,85,86,87,88,89,90,91,92,93,94,95,96,97,98,99].

To quote, with permission, from my earlier paper [107], “In much higher frequency ranges, of light, for example, dielectric properties determine the refractive index, optical properties, and thus spectra of materials [88], because the polarization of electron orbitals determines how atoms absorb and radiate electromagnetic energy. Spectra are so varied that they are used as fingerprints to identify molecules [88,90,91,93,94,95]. ***Spectral properties are as diverse as molecules and obviously cannot be described by a single constant refractive index***”.

Many interactions of light and materials cannot be described at all by dielectric constants. Dielectric constants are useful only when field strengths are small enough so polarization is a linear phenomenon, linearly dependent on field strength. Some of the most interesting applications of electrodynamics involve nonlinear, field dependent polarization [100,101,102,103,104,105,106].

**The dielectric dilemma** is clear: nonlinearities, spectra, and diverse dielectric behavior cannot be described by a single dielectric constant, but Maxwell’s equations use a single dielectric constant, as they are usually written.

When a dielectric is complex, polarization and dielectric behavior need to be described by a functional, and the very form of the Maxwell equations changes. The detailed properties of polarization need to be known and described under the range of conditions in which polarization has significant effects. Polarization is rarely known that well experimentally under all the conditions of interest. Theoretical models or simulations of that scope that actually fit the range of experimental data with one set of unchanging parameters are even scarcer.

**Maxwell’s equations with a single dielectric constant remain of great importance**, however unrealistic the approximation, because that is how they have been taught [4,42,47,49,50,110,111,112,113]; [106] is the exception) ever since the equations were formulated [42,114,115,116].

Students often remain unaware of the complex properties of the polarization of matter until they become scientists trying to use electrodynamics in applications. As scientists, they face a dielectric dilemma [107]. Too little is often known of polarization to make the Maxwell equations useful in applications demanding precise understanding.

Of course, if no measurements are available, it is much better to assume a dielectric constant that is a single real positive number (>1) than to ignore the dielectric altogether.

**Importance of the dielectric assumption for biology and chemistry.** Much of chemistry, and all of biochemistry, and biology occur in liquids, so classical Maxwell equations with their constant $\varepsilon_{r}$ are inappropriate for these applications, which involve a significant fraction of all science, judging by the relative budgets of biological and medical science compared to those of the other sciences, even semiconductor technology. Of course, science depends on oversimplifications, and approximations, but the scale of the variation of polarization is breathtaking and often overlooked. The effective dielectric coefficient of proteins ranges from 80 in normal low frequency linear measurements, to 2 **on the time scale of molecular dynamics simulations.** Atomic scale simulations are absolutely required to deal with the crucial fact of molecular biology: replacing a handful of atoms—sometimes even one atom, when ionization of an acid or base is involved for example—has dramatic macroscopic effects on the functions of proteins (think ion channels), cells (that depend on the behavior of ion channels, like nerve and skeletal muscle fibers), tissues (that depend on the behavior of cells), and organs (like the heart, that depend crucially on the behavior of individual ion channels. Clinical reality shows that disturbances of one ion channel (the *herg* channel of the heart) leads to irreversible arrhythmias and death in large numbers of people. Many of the young adults who die of “heart attacks” are likely to be victim of drug binding to the *herg* channel that is “addicted to… cocaine, alcohol, and ether” [117] reviewed recently in a clinical context in [117,118,119].

**Dielectric properties of solids.** When the Maxwell equations were first written in modern form, by Heaviside [120] more than anyone else, time domain measurements of solids were slower than say 0.1 s, and a constant dielectric constant was a good place to begin (although even then the mixing of physical meaning of permittivity of a vacuum and permittivity of a matter was likely to be a source of confusion and thus should have caused concern. However, modern science routinely measures and uses electrical currents on the time scale of 0.01 ns. Modern science includes optical measurements and measurements even of X-ray quite routinely. Modern optics [100,101,102,103,104,105,106] exploits field dependent phenomena as its essential tool. One cannot expect a theory designed to work at 0.1 s to work on the modern time scale extending to $10^{-16}$ s for electrodynamics and much smaller times, e.g., $10^{-20}$ s for x-rays, used to determine protein structure.

The classical Maxwell equations therefore must be revised, into quite different form in fact, when the dielectric constant is not constant, that is to say, when the polarization cannot be described by a single real positive number $\varepsilon_{r}>1$.

In fact, the description of polarization by a single positive real number is almost never an adequate representation of the properties of real systems [29,57,58,59,60,61,62,63,64,65,66,67,68,69,70,71,72,73,74,75,76,77,78,79,80,81,82,83,84,85,86,87,88,89,90,91,92,93,94,95,96,97,98,99,100,101,102,103,104,105,106,107,108,121,122,123]. The reformulation of the Maxwell equations for nonconstant $\varepsilon_{r}-1\;$ will produce equations with very different mathematical form, in general requiring convolutions in the time domain.

Of course, as stated before, and restated here for emphasis, when nothing is known experimentally about polarization P, it is better to use a dielectric description with $\varepsilon_{r}$ constant, than with no polarization P≅0, at all.

**Maxwell core equations are not constitutive equations.** The core equations contain no parameters describing matter. The core Maxwell equations involve only two parameters, and those are parameters of space, not matter: the magnetic parameter (i.e., permeability of space) $\mu_{0}$, and electric parameter (permittivity of space) $\varepsilon_{0}$, and perhaps the speed of light $c=1/\sqrt{\mu \varepsilon_{0}}.$ These parameters are true constants within the accuracy (~10^−8^) of measurements of the fine structure constant α of quantum electrodynamics. They are universal field equations true everywhere, in the vacuum of space and in matter, including in the vacuum within and between atoms.

The core Maxwell equations may seem to be quite useless without a specific description of material charge. Indeed, they are useless (without a specific descriptin of matter) if the goal is a complete description of electrodynamics.

If the goal is to describe the properties of (total) current, however, the core Maxwell equations are remarkably useful, even without knowledge of constitutive properties. It is important to understand that the use of the Maxwell Ampere law to derive conservation of total is not restricted to systems where magnetic properties are significant. Conservation of total current is as accurate and general as the Maxwell equations themselves, extending to a component of the Schrödinger equation inside atoms. The result is independent of material properties as its derivation makes clear.

**Derivation of Conservation of Total Current.** We start with the Maxwell equations that include Ampere’s Law as Maxwell formulated it

(8) $$\frac{1}{\mu_{o}}\;curl\;B=J_{total}=J\;+\;\overset{\begin{array}{c} \mathrm{Vacuum}\;\\ \mathrm{Displacement}\;\\ \mathrm{Current} \end{array}}{\overbrace{\varepsilon_{0}\frac{\partial E}{\partial t}}}$$

(9) $$\;J=\underset{\begin{array}{c} \mathrm{Material}\\ \mathrm{Displacement}\\ \mathrm{Current} \end{array}}{\underbrace{\left(\varepsilon_{r}-1\right)\varepsilon_{0\;}\frac{\partial E}{\partial t}}}+J_{everything\;else}$$

B is the magnetic field $\left(\varepsilon_{r}-1\right)\varepsilon_{0\;}\partial E/\partial t$ is the polarization of idealized dielectrics and is separated in Equation (9) as in much of the literature. $\;J_{everything\;else}$ includes migration of charge carried by anything from atoms, to molecules, to components of proteins, to quasi particles like holes and ‘electrons’ of semiconductors. $\;J_{everything\;else}$ also includes all material polarization, no matter how fast, transient and small. Thus, $\;J_{everything\;else}$ includes the classically defined dielectric current $\left(\varepsilon_{r}-1\right)\varepsilon_{0\;}\partial E/\partial t$.

The divergence of the curl is always zero, as discussed in any text on vector calculus, and is easy to show by simple substitution of the explicit forms in terms of derivatives with respect to Cartesian coordinates [27,28]. Readers unfamiliar with vector calculus, or skeptical of the generality of the results, are urged to perform the substitution so they will see the cancellation of terms, and be convinced of this important result: ***conservation of total current is as general as Maxwell’s equations themselves.***

Conservation of Total Current

(10) $$div\;\left(\;\overset{Current}{\overbrace{J+\varepsilon_{0\;}\frac{\partial E}{\partial t}}}\;\right)=0$$

because

(11) $$div\left(\frac{1}{\mu_{o}}\;curl\;B\right)=0$$

An incomplete solution of the equations for the electric field E(x,y, z|t) is helpful for physical understanding (although much more is involved than the solution Equation (12) displays).

(12) $$E\left(x,y,\;z|t\right)=-\frac{1}{\varepsilon_{0}}\;\int_{0}^{t}J\left(x,y,\;z|\tau \right)\;d\tau \;$$

The equation shows that that the electric field can assume the value needed to move the atoms so that the total current is preserved. The electric field helps determine the force on atoms and thus the movement of the atoms. The Maxwell equations guarantee that the atoms move just enough to conserve total current. This is how the Maxwell equations express themselves on an atomic scale so the conservation of total current is true on all scales.

Note that in this equation, the electric field is an output. E(x,y, z|t) is ***not*** assumed. It is an output of the analysis. E(x,y, z|t) is the result of the integration of the Maxwell equation and so depends on ‘everything’.

**In networks of circuit components,**E(x,y, z|t) is different in different components as determined by the global physics and structure of the network as well as local properties. Local properties themselves do not determine the electric field or the flow of total current.

As stated in many of my earlier papers, the electric field is both global and local. This reality is most vivid in one dimensional networks where components are in series. Currents $\;J_{total}$ are equal in every component of a series system, at all times and in all conditions. The current in one component depends on the current in another. ***The microphysics of conduction in one component does not in itself determine the current flow through that component, despite our local intuition which might suggest otherwise.***

**Figure 3:**
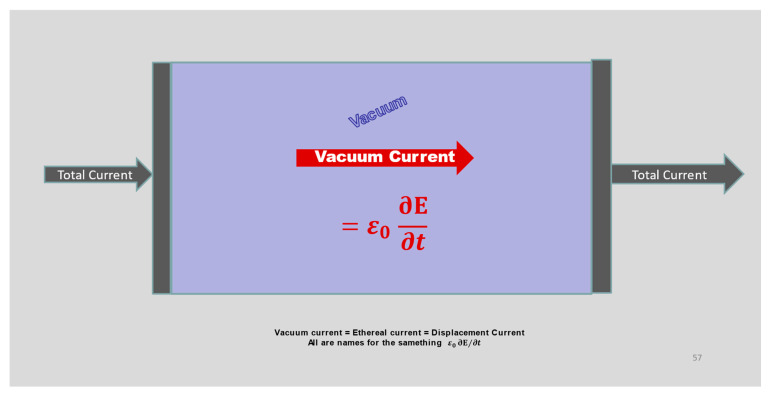
The vacuum capacitor illustrates the equality of total current.

Reference [124] discusses this property of series systems in detail, showing how the physics of each component consists of both the local microphysics specific to that component, and also the polarization of the vacuum, the displacement current $\varepsilon_{0\;}\partial E/\partial t$. Figure 3 of [124], and its discussion, show how E(x,y, z|t) varies in wires, resistors, capacitors, diodes, ionic solutions. E(x,y, z|t) varies in every component but $\;J_{total}\left(t\right)$ is always the same everywhere, at any time (although it varies with time), because the components are in series. The currents are equal in the components of the series circuit of Figure 3 [124], at all times and in all conditions, because the Maxwell equations produce the E(x,y, z|t) field, the material currents and fluxes, and the ‘vacuum’ displacement current $\varepsilon_{0\;}\partial E/\partial t$ needed to conserve current $\;J_{total}$, no matter what are the local microphysics of conduction or polarization [61,123,124,125], no matter what the dielectric current is in its classical approximation $\left(\varepsilon_{r}-1\right)\varepsilon_{0\;}\partial E/\partial t$.

**Constitutive theory of charged matter is rather similar to the constitutive theory of mass**. Polarization can be described by constitutive equations. The stress strain relations of solids are a constitutive theory of mass, as are the stress strain relations of complex fluids [53,54,55,56]. They describe how density varies with pressure and other mechanical forces. The variation of charge density with electric field can be described in the tradition of fluid mechanics. The variation of charge density with electric field can be described the way the variation of mass density with mechanical force is customarily described.

**Polarization**. The distribution and amount of charge in matter varies with the electric field. Charge is said to polarize in the electric field. The phrase “to polarize” means to change the density (and distribution of density) of charge. Something that does not polarize is something in which the density of and distribution of charge does not change when electrical forces change.

The charge in solid matter polarizes. So does the charge in liquids. The solvent molecules and the solute molecules in liquids can change their orientation and their internal distribution of charge as the electric field changes. They polarize. They make a contribution to the classical Maxwell polarization field. The previously cited literature on the dielectric properties of matter is devoted to the measurement, description and analysis of this polarization, including [29,57,58,59,60,61,62,63,64,65,66,67,68,69,70,71,72,73,74,75,76,77,78,79,80,81,82,83,84,85,86,87,88,89,90,91,92,93,94,95,96,97,98,99,100,101,102,103,104,105,106,107,108,121,122,123]. The polarization cannot be adequately described by a single dielectric constant $\varepsilon_{r}$, by one positive real number $\varepsilon_{r}>1$.

The name ‘concentration polarization’ is used to describe quite a different phenomenon in the modern and classical literature of ionic solutions. Readers unfamiliar with the use of the word ‘polarization’ in the phrase ‘concentration polarization’ are urged to look at the modern literature [126,127,128,129] lest they confuse concentration polarization (of ionic concentrations) with the polarization of charge described by Maxwell’s P field, see Equation (6).

Interestingly, Hodgkin, Huxley, and Katz [130], in their paper that is the foundation of modern electrophysiology, leave out the modifier ‘concentration’ (of the phrase ‘concentration polarization’) and say just ‘polarization’ thus making it easy for electrophysiologists to confuse dielectric and concentration polarization. History of science texts explain how the various meanings of the word ‘polarization’ [42,48,114,131] arose although they underemphasize the confusion that resulted, and still is common, in my experience and opinion.

**Molecular Polarization.** Molecules polarize in complex, time and field dependent ways as implied by the long earlier discussion of the inadequacy of the classical approximation of a dielectric constant $\varepsilon_{r}$ as a single real positive real number $\varepsilon_{r}>1$. So do atoms, and of course aggregates of molecules, as reported in the literature of impedance, dielectric and molecular spectroscopy [29,57,58,59,60,61,62,63,64,65,66,67,68,69,70,71,72,73,74,75,76,77,78,79,80,81,82,83,84,85,86,87,88,89,90,91,92,93,94,95,96,97,98,99,100,101,102,103,104,105,106,107,108,121,122,123]. Polarization of proteins is one kind of the conformation changes of proteins used so widely to describe their function. Conformation changes occur over an enormous range of times scales in proteins. So does polarization.

**Forces Change Distributions of Mass and Charge.** It is obvious that a mechanical force applied to a mechanical system changes the distribution of mass. It should be obvious that electrical force applied to a system of charges changes the distribution of charge.

As the electric field changes, forces change the amount and location of charge, much as a mechanical forces (stress) change (strain) the amount and location of mass. 

A description of the change of distribution of mass is likely to be quite specific to the system being studied. The ***description will depend on the structure*** within which the matter (and thus the field equations) are embedded, and on the boundary conditions that describe the properties of the boundaries and the locations and properties of the structures. 

Generalities are likely to be too vague to be very useful in applications because applications almost always depend on the shape of the structure containing the force fields and the boundary conditions that describe the physics that occurs at the boundaries, as well as how the structures and physics change with conditions. ***There is little engineering without structure. There is no biology without structure.*** The structures constrain the system. The structure provides a framework on which the designer—whether engineers or evolution— hangs the boundary conditions. The boundary conditions are, the equations that link structure and physics. Boundary conditions also define the inputs and outputs of engineering devices and of the evolutionary devices —like ion channels—that fulfill the engineering definition of devices.

It should be obvious that an electrical force applied to a charged system changes the distribution of charge. Additionally, a description of the change of charge distribution is likely to be quite specific to the system being studied for the same reasons. The change of charge with the electric field (i.e., with electric forces) is used by engineers whenever they use a capacitor. The change of charge with fields is used by biology whenever evolution uses a membrane. All membranes have large capacitances. Describing the change of charge distribution with field is an essential part of describing how engineering or biological systems work just as describing the change of mass distribution with pressure is an essential part of describing how hydrodynamic systems work, whether they are in engineered devices or in the fluid mechanics of the kidney designed by evolution [132,133,134]. Of course, the Maxwell equations show that charge changes when the electric field changes. All engineering, all biology, and all physics involve the change in charge with the electric field as seen most precisely in the Core Maxwell equations. In the more familiar classical Maxwell equations all physics involves the change in charge with electrical forces because the dielectric constant $\varepsilon_{r}>1$. The Maxwell differential equations are needed to describe electrodynamics because the electric field changes with charge, as the charge density varies with the electric field. Algebraic equations cannot describe such interactions.

**Complex fluids.** The change in distribution of mass can be described in many ways. The stress strain formalism of complex fluids is a powerful and general approach [135,136,137,138,139,140]. In its variational form [53,54,55,56,139,141,142,143], the stress strain formalism accommodates diffusion and convection that are so important in liquids. The variational form allows the large movements of mass and charge produced by convection and diffusion in liquids to be described, along with the much smaller movements of mass and charge associated with the elasticity of solids. The general literature can be accessed through the literature of the variational treatment [53,54,55,56,139,141,142,143].

The stress strain formalism of polarization can accommodate the diffusion, migration, and convection of charge in solutions in much the same way

## 3. Results

**Polarization can be treated as the stress strain relation of charge** (see Equations (3.1)–(3.5) of ref. [144]). In its variational form, the stress strain formalism accommodates diffusion and convection that are so important in liquids, yielding the classical Poisson Nernst Planck equations in special cases [53,54,55,56] important in applications ranging from ions in water solutions, ions in protein channels, to ions in gases [145] and plasmas [146,147,148], to holes and electrons that are the quasi-ions of the semiconductors of our computers and smartphones [149,150,151,152,153].

It is obvious that stress strain relations are hard to summarize. They usually involve a multitude of parameters chosen to describe the specific properties that determine the deformation of matter. They can be nonlinear and sometimes involve multiple types of forces each of which is customarily described by its own disjoint field theory with partial differential equations and boundary conditions.

It should be obvious that the stress strain relations of charge will be at least as hard to summarize as those of mass. Those polarization properties will involve a multitude of parameters chosen to describe the deformation of distribution of charge by electric forces. A single dielectric constant $\varepsilon_{r}>1$ will hardly ever be adequate, despite its historical provenance [99,107]. Of course, when nothing is known experimentally about polarization, it is better to use a dielectric description with a single constant $\;\varepsilon_{r}>1$ than nothing at all.

Once polarization is separated from the core Maxwell equations, it is clear that the core equations are fundamental, universal and as exact as any in science [29]. Without polarization, the Maxwell core equations have only two constants and these are ***not*** adjustable. These constants are known to be just that… constant. They do not change in any known experimental conditions. They are determined with great precision by any two of the experimentally determined properties, the electrical constant $\varepsilon_{0}$ (the permittivity of free space), the magnetic constant $\mu_{0}$ (the permeability of free space), and speed of light c.

**Maxwell equations require boundary conditions on a finite structure**. Maxwell equations of electrodynamics are partial differential equations that require boundary conditions specified on a finite–***not infinite***–structure, called a domain in mathematics. Boundary conditions are discussed in the mathematics literature as part of the Helmholtz decomposition, using the Hodge decomposition of (more) pure mathematics, to establish the Helmholtz theorem. The necessity of boundary conditions is a central result in the classical theory of fields documented in classical and modern textbooks of theoretical physics (e.g., [154,155] and applied mathematics). Reference [28] spends many pages illustrating the role of boundary conditions in a variety of partial differential equations, showing those that are consistent or inconsistent with particular differential operators. The physically oriented discussion in Appendix B (p. 555) of [113] is particularly useful in the electrical context. 

**Boundary conditions in electrodynamics can be delicate**. The requirements for the distribution of permanent charge in space are particularly delicate as described by Griffiths: to paraphrase p. 556, if the divergence of the electric field and the curl of the fields involved decrease more rapidly than $1/r^{2}$ as r→∞, all integrals required converge. This argument deals rigorously with the charge density within the system. However, the boundaries of the system are different. They usually contain charge themselves. The vector operatorsact on charge on the boundaries, and are not just functions of the charge density within the system. The convergence properties thus also depend on the structure of the boundaries and the detailed distribution of charge on the boundary. Many parameters are needed to specify real boundaries—they are rarely simple spheres. This somewhat abstract mathematics becomes quite concrete when one realizes that the inputs and outputs present in all the devices of our technology are boundary conditions. The input and output impedance of electronic devices provide the detailed description of charge on the boundaries of our electronic devices. As anyone who has actually built a circuit knows, it is essential to respect the properties and limitations imposed by the input and output impedance of devices. Indeed, the limitation in the ability of devices to provide charge at very high speeds is perhaps the most important single limitation on the speed of our electronic devices including the circuits of our video screens, computers and mobile phones.

After the boundary and conditions are specified, the size of the boundary structure, and the domain it contains, can be increased ‘to infinity’ to see if a unique boundary condition at infinity is possible, independent of shape, location and parameters. This abstract discussion is made concrete by considering a system confined within elliptical boundaries. The system will behave one way if the ellipse is a circle, and a unique boundary condition may be possible. Obviously, the system will behave very differently if the ellipse is very narrow and behavior will change (in general) depending on which direction is the narrow one in the ellipse. A single useful general boundary condition is unlikely to be able to describe such diversity of behavior of narrow elliptical systems. The system cannot be described by a uniform boundary condition ‘at infinity’.

The reader is reminded that boundary conditions at infinity can be defined precisely only by a limiting process applied to a finite boundary. The problem is solved with the finite boundary, and then the boundary is allowed to move to infinity. There is no other way to define boundary conditions ‘at infinity’. Boundary conditions ‘at infinity’ always involve the limit of a finite boundary condition.

‘Infinity’ is not a number. It does not satisfy the equations of arithmetic as defined by the axioms of the field theory of complex numbers. ***‘Infinity’ is defined by a limiting process, not by the properties of a number.***

The behavior of the shape and parameters of the boundary structure need to be specified as the structure gets larger and larger, reaching towards infinity in the limiting process. ***Different behaviors will produce qualitatively different results, as in any nonuniform limiting process,*** including those so familiar from the theory of asymptotic series and perturbation approximations to physical systems [156]. Indeed, issues of nonuniform behavior are central to most applications of asymptotics in science.

It is irritating but necessary to remember that limit processes are subtle as well as complex. If several variables are involved, each may go to infinity in different ways. Just consider what is meant by the limit of a cylinder (in cylindrical coordinate systems if you wish) at infinity. Obviously, the limit as the length variable goes to infinity as the radial variable is fixed is one thing (a line) but the limit as the length variable goes to infinity as the radial variable also goes to infinity is something else again. Indeed, if the radial variable goes to infinity faster than the longitudinal variable goes to infinity, the resulting system is all space (not a line at all, three dimensions vs. one dimension) and to make things more complicated, the properties of the three dimensional space are qualitatively different depending on how the third variable (the angular variable) goes to infinity! Each of these possibilities will produce different geometry at infinity and so each will produce different boundary conditions, even if the physics at all the boundaries is the same. Of course, the real situation might be much more complex. The physics itself might differ from coordinate to coordinate or might vary with location.

**Defining infinity.** It is obvious then that defining a system at infinity is a specific ‘constitutive’ problem different for different systems. To reiterate this crucial point, ‘infinity’ is not a number satisfying the axioms of the field theory of arithmetic. ‘Infinity’ is an idea in science, a limiting process in mathematics.

It seems hopeless to say anything general about boundaries (except conservation laws that are likely to be too general to describe the specific behavior of particular systems that make them worth studying). Indeed, if the particular systems are designed by engineers or by evolution the complexity of possible boundary structures and conditions is likely to be exploited to use the diversity of behaviors for specific functions. After all the inputs and outputs of engineering systems are often Dirichlet boundary conditions setting the electrical potential far away from the system. Of course, the input and the outputs that characterize most engineering systems and all devices are not the same and are not at the same place. The mathematician and physicist is confronted by the need to understand spatially nonuniform (i.e., ‘mixed’) boundary conditions whenever devices are involved [132,133,157]. (Power supplies require other distinct far field boundary conditions: almost all devices require power supplies to function. The equilibrium or near equilibrium so thoroughly studied in classical thermodynamics and physical chemistry cannot describe the devices that make up our technology, for these reasons, in almost all situations in which these devices actually perform their functions.)

It is clear then that electrodynamic phenomena‘at infinity’ are so diverse in practical applications that they cannot be specified in a general way. An illuminating example is the behavior of light at infinity, discussed in detail later. The phenomena of electrodynamics include light that propagates from the edge of the universe over billions of years. The phenomena of electrodynamics also include decaying phenomena of electrostatics determined by (for example) Coulomb’s law.

Statistical mechanics and thermodynamics of matter must include electrodynamics because charges are everywhere in matter. As we have seen, interactions of even (nominally) uncharged atoms like argon involve transient charges fluctuating unavoidably as quantum and thermal dynamics say they must. The Maxwell equations that specify the behavior of these changes accurately describe e the range of electrodynamic phenomena involved on the time scales of atomic motion. Statistical mechanics and thermodynamics must satisfy the Maxwell equations. Thus, statistical mechanics and thermodynamics must be specified in the finite domains required to define electrodynamics and the Maxwell partial differential equations. That is a main point of this paper.

We turn now to a more detailed presentation of these same issues.

**Maxwell Equations are true on all scales**. The Maxwell equations have properties that are not common in scientific theories, and these need to be understood explicitly as we seek firm foundations for our theories and simulations.

For example, the Maxwell equations in general, and the Poisson version of Gauss’s law (Maxwell’s first equation Equation (2)) are often treated as averaged or mean field theory equations in my experience, perhaps because of the enormous variations of potential (say 1 electron-volt or 40 times the thermal energy) in a few picoseconds in atomic scale systems, as resolved in the simulations of molecular dynamics. Faced with this much variation, scientists are likely to think that equations describing potential must be averages. That is not true [100,101,102,103,104,105,106]. ***The Maxwell equations are not averages.*** They describe potential as a function of time on the atomic time scale of 10^−15^ s and much faster, even much faster than the electron time scale of say 10^−19^ s of quantum chemistry. The Core Maxwell equations are not mean field theories or averaged in any sense.

Mean field or low resolution models of charge may indeed be averaged meaningfully in some applications. However, the averaging occurs ***within the models*** of J and ρ, not in the Maxwell equations themselves. Maxwell equations—whether core Equations (2)–(5) or classical Figure 2—themselves are not averaged. For example, averaging is usually found in the theories and simulations of polarization, e.g., it occurs in the stress strain theories of the distribution of charge and matter we have discussed [144]. Indeed, if polarization is described in its full complexity of time and field dependence [29,100,101,102,103,104,105,106,121,122,123], the mathematical structure of the classical Maxwell equations changes. The form of the classical Maxwell equations changes in that case. The phenomena describe by an over simplified single real dielectric constant $\varepsilon_{r}>1$ are replaced (speaking roughly) by convolutions, and the electrodynamic equations may have to become integro differential equations to accommodate the complexity of real dielectric and polarization behavior.

Here is where multiscale discussions enter that are important to constructing classical statistical mechanics. Much of the variational treatment of complex fluids was designed to deal with these multiscale issues [139,158,159,160,161,162,163,164] and readers are referred to the literature for further discussion [53,54,55,56,135,136,137,138,139,140]. The location, type, and role of boundary conditions is an important topic in the theory of complex fluids.

**Maxwell Equations in a vacuum.** Many important properties of electrodynamics are apparent in a simple system, when the Maxwell equations are applied to a vacuum where J=0**,** $\tilde{J}=0$ and $\rho =0,\;\rho_{free}=0\;.$ Indeed, this application was historically central to the development of Maxwell’s theory of electricity [115]. In the vacuum, described mathematically that way, the source of the magnetic field B is the ethereal displacement current $\varepsilon_{0}\partial E/\partial t$ (because div B=0). Currents and perhaps charges found on structures that form the boundaries of the vacuum region can also be sources of the magnetic field.

The ethereal displacement current $\varepsilon_{0}\partial E/\partial t\;$ is universally present in matter and in a vacuum, because it arises from the relativistic invariance of charge with velocity, as described in textbooks of special relativity [165], in Einstein’s original paper [25,166], or memorably at several places including Section 13-6 Feynman’s textbook volume 2 of [4].

**Ethereal Currents.** The implications of the ethereal term $\varepsilon_{0}\partial E/\partial t$ are profound. The Maxwell equations involve (total) current flow and E fields in all of space, and cannot be confined to atoms in atomic resolution simulations. The Maxwell equations describe electric fields in discrete simulations of atoms because $\varepsilon_{0}\partial E/\partial t$ exists everywhere in those simulations, as it does everywhere in space, even if all charges are confined to atoms. Derivations of statistical mechanics of atoms must include the same realities as simulations of the electrodynamics of atoms and so are subject to the same issues. In plain English, electric fields and ‘currents’ exist in between atoms and help determine the forces between atoms. They must be included in simulations that are usually thought to involve entirely discrete variables.

The Maxwell equations are not confined to continuum descriptions of charge. They also describe the motion of charged atoms in a continuum. Simulations of charged atoms in a continuum include currents carried by the charged atoms. Simulations of charged atoms in a continuum also include the currents in the continuum. If the ‘outside the atoms’ currents are ignored, the simulations cannot satisfy Equations (2)–(5) everywhere and at every time. If outside the atoms currents are ignored, the system will not follow the laws of electrodynamics. If outside the atoms currents are ignored, the systems will not follow the laws of quantum dynamics (e.g., the Schrödinger equation) because quantum dynamics embody electrodynamics (at the least in the variable V in the usual formulation of the Schrödinger equation). Derivations of statistical mechanics must include the same realities of electrodynamic fields as simulations of atoms and so are subject to the same issues if statistical mechanics and electrodynamics are to be compatible and consistent.

To summarize this section: $\varepsilon_{0}\partial E/\partial t$ cannot be avoided even in atomic simulations.

This fact often surprises colleagues used to thinking of electricity as the properties of charged atoms, and their movement. However, electricity is much more than charges and their movement. It includes all the properties of light and electromagnetic radiation everywhere. If my colleagues think of electricity as the properties of charged atoms, they have difficulty understanding the properties of the space between stars where there are no atoms, but where electricity (and magnetism) combine to allow starlight to reach the earth in a heavenly illumination.

**Electricity always includes the ethereal displacement current term**$\varepsilon_{0}\partial E/\partial t$. Without $\varepsilon_{0}\partial E/\partial t$, there is no source for curl B in a vacuum or in the space between atoms devoid of mass between or within atoms (assuming no currents on boundary structures), and light cannot exist or propagate.

The ethereal term does not depend on the properties of matter. It in fact is a property of space, not matter, arising from the fact that charge is Lorentz invariant in any locally inertial reference frame as discussed in textbooks of special relativity, in Einstein’s original paper on electrodynamics [25,166], or memorably in Section 13-6 of Feynman’s textbook volume 2 of [4]. Charge (unlike length, time, and relativistic mass) does not change as charges move, no matter how fast they move, even if they move at speeds close to the speed of light (as in the synchrotrons of a say 7 gigavolt advanced photon sources used to generate X-ray to analyze the structure of proteins).

**Electrodynamics requires differential equations**. The existence of the ethereal current $\varepsilon_{0}\partial E/\partial t$ means that any description of electrodynamics must include a partial derivative with respect to time, usually in the form of the Maxwell Equations (2)–(5) or Figure 2. The Maxwell equations are partial differential equations and so they cannot be computed, even approximately, without boundary conditions on their limiting structures, and initial conditions. In the language of mathematics, the solutions to the equations do not exist without boundary and initial conditions as shown by the literature of Helmholtz and Hodge decomposition, and the Helmholtz theorem presented in that literature. This is an issue of mathematics not physics and the reader is reminded that numerical computations (with known error bounds) reveal these issues clearly without the burden of abstraction required by the existence theorems of the Helmholtz and Hodge decomposition. Griffiths [113] Appendix B (p. 555) does a particularly good job of explaining these mathematical issues in a physical context, in my view.

**Electrodynamics and the Maxwell Equations are relevant to biology.** It is natural for biologists and biochemists to think that the previous discussion is irrelevant to their concerns. Existence properties of partial differential equations is not of major interest to most of them.

One might hope that ethereal displacement currents are small and so can be ignored. However, that is not the case as the simplest estimates show, and as can be measured in every simulation of molecular dynamics. Those simulations always include atomic time scales in which the ethereal current is large because ∂E/∂t is so large in atomic scale simulations, with electrical potentials varying something like 0.5 volts in 10^−12^ s. The variation in electrical energy is some 20× the thermal energy $k_{B}T$. Indeed, Langevin and Brownian models of thermal motion are often used as supplements to all-atom molecular dynamics. Those coarse grained Langevin and Brownian models include a noise term that is a Brownian stochastic process in the language of probability theory and have infinite variation, in the language of mathematics, which means that they have infinite velocity. The trajectories of a Brownian stochastic process cross a boundary an infinite number of times in any time interval however brief [167,168,169]. While it is not clear how to compute the ethereal current of charges moving this way at infinite velocity [169], it is clear that the ethereal current of a process with infinite velocity cannot be small. Indeed, it is quite likely to be large, beyond easy comprehension.

The idea of an ethereal current should not be strange. The concept of ethereal current arises naturally in high school physics (Figure 3). It is implicit in most elementary discussions of capacitors in which the charge $Q_{cap}=C_{cap}V$ and current $I_{cap}=C_{cap}\partial V/\partial t$. The idealized capacitors most of us studied in elementary physics classes, often as teenagers in high schools, include a large current that flows in the empty space between the plates of the capacitor. That current is the ethereal current. No material charge exists or flows there. The $\varepsilon_{0}\partial E/\partial t$ term is in fact the only current between the plates of a vacuum capacitor. The ethereal current is always ***exactly equal*** to the total current flow in the wires on either side of the capacitor, because total current is conserved exactly by Maxwell’s equations [170].

A vacuum capacitor may seem an artificial schoolchild example, although not to those of us who have wired up circuits with capacitors or to the thousands of circuit designers who include them in the many billions of circuits in our computers. Additionally, systems certainly exist for which $\varepsilon_{0}\partial E/\partial t$ is unimportant, e.g., in many systems in which ∂E/∂t=0. However, the vacuum capacitance is crucial in the atomic scale systems that are studied in statistical mechanics because atoms move quickly on the atomic scales of time and distance.

The ethereal current is almost never small for atomic scale systems, even at temperatures near absolute zero because atomic motion (and thus motion of charges) persists even at those temperatures. The ethereal current $\varepsilon_{0}\partial E/\partial t$ cannot be safely ignored in simulations or derivations of statistical mechanics that involve the atomic scale. 

It should be clearly noted that including the ethereal current $\varepsilon_{0}\partial E/\partial t$ can simplify qualitative understanding because it helps guarantee the exact conservation of total current, or Kirchhoff’s law in circuits, or the equality of total current inside devices and series systems. The conservation of total current provides easy understanding of the forces between atoms that do not collide. Conservation of total current is much easier to understand if the ethereal current $\varepsilon_{0}\partial E/\partial t$ is included explicitly in our thinking.

**Defining Infinity**. Another issue seems abstruse mathematics, but is not. The is sue is what happens in atomic scale systems as they get larger and larger. What happens in statistical mechanics ‘at infinity’.

Defining infinity is not quite the arcane point of pure mathematics it might seem to be. In fact, the idea of ‘boundary conditions at infinity’ is useful only if it has a unique meaning independent of the details of the system. ‘Boundary conditions at infinity’ are useful only if that phrase defines a wide class of structures far away from the system of interest, ***in which the details of the structures are unimportant*** because they are lost in the blur and haze of distance, as the details of the structure are lost in the word ‘infinity’.

If different structures produce different boundary conditions when the structures are far away, a single word and equation ‘at infinity’ will not be able to describe the resulting range of behaviors. In fact, ‘infinity’ cannot be defined in a unique way from the Maxwell equations themselves as the following example shows.

Consider two subsets of the Maxwell equations. Consider an electrostatic problem, with all charge in a finite region. Coulomb’s law Equation (1) can then be used to compute electrical forces. Magnetic forces do not exist (because it is a static problem). Infinity can be defined easily and uniquely and the potential or electric field at infinity is zero (in electrostatic systems like this with charges all in one region). 

If charges are not confined to one region, convergence must be considered. If the charges are dipoles, quadrupoles, or other higher order terms, convergence is assured if the density of charge is uniform in space. (Of course, if the density increases with distance, convergence issues may arise as discussed previously where literature references are given.) However, if the charge is monopoles, and with uniform density in space, the integrals needed to define forces do not converge. This is not a mathematical artifact. It is a physical reality that the forces depend on the size ***and shape*** of the system, and must be calculated that way if the charge density is uniform. Many solids have uniform charge density particularly in the idealizations considered in the models of textbooks. Those must include the properties of the boundaries of those system, for example, the charge density on the boundaries, even as the systems grow large. Convergence conventions of classical physics and physical chemistry are unlikely then to give results that actually satisfy the Maxwell equations, i.e., Gauss’ law because those conventions skirt these issues of convergence, to put the matter politely.

Now, consider a different structure described by Maxwell equations in which wave properties predominate in a pure vacuum without matter. Two relevant wave equations in this domain are derived in textbooks of electrodynamics and discussed in [171].

(13) $$\mu_{0}\varepsilon_{0}\frac{\partial^{2}E}{\partial t^{2}}-\nabla^{2}E=0$$

and

(14) $$\mu_{0}\varepsilon_{0}\frac{\partial^{2}B}{\partial t^{2}}-\nabla^{2}B=0$$

The solutions to these equations do not go to zero at infinity. In fact, these solutions never remain close to zero. The solutions describe light waves that propagate forever, as light actually does propagate over billions of light years of distance, from galaxies at the edge of the observable universe for very long times. Specifically, astronomers tell us that tthe light from the galaxy **GN-z11** started soon after the universe began some 1.3 × 10^10^ years ago billions of light years from the earth where we observe it.

It is instructive to consider boundary conditions of waves in a little more detail. If the wave occurs in a vacuum its speed of propagation is the speed of light $c=1/\sqrt{\mu_{0}\varepsilon_{0}}$. Specifying the boundary condition at infinity then involves two limiting processes, one in x,  x→∞ the other in t,  t→∞ . The limit is not uniform but depends on the way x and t vary as they go to infinity. If x and t to infinity at the same rate, at the speed c, the boundary value can be any value of the waveform that propagates. If the waveform propagates more slowly than c, because the wave is moving at velocity 𝕔 through a material like the glass of a lens instead of through a vacuum, then a zero boundary condition at infinity is possible. If the waveform moves more quickly than 𝕔 through the material, the boundary condition at infinity is not likely to be zero.

The qualitative property of the boundary at infinity depends on the speed of propagation, on the structure of the boundary, and the physical properties (i.e., boundary equations) of the boundary structure.

Atomic simulations involve such rapid motions that wave terms cannot be neglected. Indeed, they are responsible for the optical properties of the simulated system. The simulated systems form an important part of applied physics. They are studied extensively in experimentation and applications in physics, chemistry, technology, and biochemistry and molecular biology, even biology itself.

It is clear then that atomic simulations require complex boundary conditions if they are to be compatible with the Maxwell equations.

## 4. Discussion

The analysis of ‘at infinity’ shows in a mathematically precise way that the Maxwell equations do not have a single set of boundary conditions ‘at infinity’ as shown in careful mathematical analysis (cited above) and is also obvious physically. Rather, each application of the Maxwell equations requires an explicit definition of confining (as well as internal) structures and the boundary conditions on those structures. It also requires a statement of how structures and conditions vary as the system gets bigger and bigger, to infinity. One description of structures and boundary conditions cannot be enough, no more at infinity than anywhere else in a system that is considered as it grows larger and larger.

Thus, any description of electrodynamic phenomena in systems get large without limit needs to specify

(1) the structure of the system
(2) the boundary conditions on the confining structure that bounds the system
(3) the change in shape of the structure as it moves ‘to infinity’
(4) the change in boundary conditions as the structure moves ‘to infinity’

**Statistical Mechanics unbounded**. What are we then to make of the fact that most treatments of statistical mechanics do not include boundary conditions?

Surely the results of these analyses must have value even if they are unable to include the Maxwell equations!

Of course, classical statistical mechanics has immense value. In my view, the classical results serve as a first model, from which to construct other more refined models. The more refined models can include structures and boundaries that are allowed to move to infinity. In many cases the properties enumerated a few paragraphs above can be stated with ado in just a few words.

In this view, classical statistical mechanics provides an admirable starting point for the iterative social process we call science. Statistical mechanics provides a first iterate for the handling of statistical properties of idealized, albeit often impossible, systems. The first iterate may itself suffice in some cases, and boundary behavior be described in a few words. Later iterations provide the improvements that allow charge and the equations that describe charge. Those equations include the structures that bound the charge and the conditions on the equations at those structures.

However, ***we must allow the scientific process to iterate if it is to improve.*** We must extend statistical mechanics to include structures and boundary conditions. We must remember that statistical mechanics without spatial bounds has logical bounds. It is not a universal set of laws. Statistical mechanics is a model that must like all other scientific models be compared to experiments. Those experiments include structures and bounds. We must not allow tradition to prevent progress.

What is clear is that boundaries must be included in the final iterates of our theories and simulations of the statistical mechanics of matter, because matter is charged. Matter is charged by the Maxwell and Schrödinger equations and they are bound to include boundary conditions. They are confined by structures that form spatial boundary conditions as are all partial differential equations.

**Statistical Mechanics within Boundaries**. The inclusion of structures and boundary conditions in statistical mechanics is likely to require extensive investigation of specific problems [172] and these will not be easy to study, judging from work in related fields for example, the theory of granular flow [173,174,175] and soft matter [140,176]. Specifics are needed because specific problems involve specific structures and specific physical properties of those structures.

The structures can be as important as the field equations themselves. It is obvious that field equations in biological systems express themselves through the hierarchy of structures that characterize life, from atomic to macroscopic scale [177].

It is just as obvious that the devices that make modern life possible are controlled by their structure, as much as by the physics that the structure controls. It is the structure of the “piston in a cylinder” that converts the combustion of gasoline into motion. The field theory of combustion is silent about the motion without the structure. It is the fantastic hierarchy of structures in our semiconductor devices that processes information as the structures control the flow of current in the branched one dimensional structures of their logic units.

Each structure needs separate investigation and general theories will tend to be less useful than one would wish. A general theory of logic units is certainly helpful. As is a general theory of internal combustion engines. However, neither is a substitute for an instruction manual, let alone a design and repair manual.

A simple example shows that boundary conditions are needed in statistical mechanics, even in imaginary systems that have no electrical forces. Consider triangular objects (‘molecules’ in a flatland [178,179]) in a two dimensional universe in a triangular domain [180]. It is obvious that if the triangles are similar, i.e., have the same shape, the triangles can lock, they can jam into an immobile array nearly crystalline in nature. This jamming can occur no matter how large the system, no matter how far away is the boundary. These issues are well recognized in the specialist literature of granular flow [173,174,175] but their remedy is unclear, not yet at hand [176,181,182], as far as I can tell. It is obvious that similar issues can arise when molecules pack together particularly at the very high number densities important in enzymes [183] and ion channels [184,185,186].

It seems necessary to consider boundaries as one tries to design a statistical mechanics of real systems even fictitious systems without electrical properties.

Meanwhile, one can proceed in an entirely different tradition, the tradition of complex fluids [53,54,55,56,135,136,137,138,139,140]. Here, field equations are used to describe each of the force fields: stress stain mechanical relations, diffusion, electrical migration, and convection. Fields are combined by a variational approach like EnVarA [53,54,55,56] that guarantees mathematical consistency of the models chosen.

The key is to always make models of specific systems—including the apparatus and setup used to study them—and then to solve those models with systematic well defined approximations that other scientists and mathematicians can verify, falsify, correct, and extend. With modern numerical and computational methods, and highly skilled mathematicians interested in these issues [187], systems as small as the voltage sensor component [188] of the protein of an ion channel [189,190,191,192,193,194,195], or as complex as the lens of the eye [196], a piece of the ‘brain’ (central nervous systems) [197,198], or systems that extend from the atomic to the macroscopic scale, like the cytochrome c oxidase enzyme of mitochondria [199] can be analyzed, although each involves many (sometimes 21) partial differential equations.

**Biology is easier than physics** in this particular case. In general, creating multiscale multifield models is a forbidding challenge, because the range of behaviors is so large when convection and diffusion move charge and mass, as well as electrodynamics. Almost anything you imagine can happen from the shock waves of supersonic transport to the frightening lightning seen every few seconds throughout hurricanes, to the smooth ohmic flow of charge in metallic resistors or in salt solutions. Additionally, this range of behavior of fields is made much larger when fields are confined within structures with special properties at the boundaries like at the inputs and outputs of the devices of our electronic technology. They too can impose a wide range of behaviors indeed.

Fortunately, one does not have to work in general if one is interested in engineering or biological systems.

Biology and engineering are rarely concerned with all possible systems. They are mostly concerned with specific systems with specific structures that behave robustly when the systems are used as they were designed, when the parameters of the system are in certain limited ranges. These systems have a purpose and that requires them to follow macroscopic rules over a substantial range of conditions.

The design of the systems of biology and engineering can make analysis easier. There is no need to study the operation of an automobile engine with water in the gas tank, or of an amplifier without a power supply. There is little need to study the behavior of dead animals, although the behavior of dead plants like trees form an important exception.

The first rule is to study the system only in the conditions in which it is known to function. Moving outside those conditions is likely to make behavior far more complex, as well as irrelevant, although perhaps useful in other ways. (Think of dead plants and wood of dead trees.)

The second rule is to focus on the function of the system and not all of its other possible behaviors. (Trees function in structures when they die and are turned into wood.) The function of a system in biology and engineering must be predictable and reasonably robust or the system is of little use. Engineers usually design systems with simple behavior so prediction is easy. Physiology shows that many biological systems also behave simply if one focuses on their function, and not on everything they might do.

Studying systems in their natural function thus makes the task of scientists and mathematicians much easier.

An important caveat is that one must know the function to study it! This is hardly a problem in engineering but it is a central problem in the history of biology. Put crudely, evolution selects systems that produce more offspring that themselves can reproduce. Knowing how a biological system aids in this process of natural selection is often difficult.

Many systems often have obvious functions, but many do not. It took centuries to determine that blood vessels circulate blood and oxygen. It is a sad fact that many of the systems of our extraordinary nervous systems process information in an unknown way. Determining these functions is a non-trivial task that has been the life’s work of generations of biologists, anatomists, and physiologists since those words were invented millennia ago and used by Aristotle [200] in ways we can recognize as physiology or anatomy in the modern sense of those words.

Many functions have been isolated and understood by now [201,202,203,204]. We know what the heart does. We know what muscle does. We know the function of the ribosome, of ion channels and so on.

Multiscale Multiphysics models that study function can take advantage of the simplifications that evolution has used once we have learned what those simplifications are. Confining models to stay on these beaten paths of physiology and anatomy focuses attention and makes possible what otherwise seems unapproachable. The Hodgkin Huxley treatment of the binary signal of nerve and muscle (now mysteriously called ‘digital’ although the signal does not involve fingers, or the numbers five or ten, or fifteen or twenty if we want to include toes among digits) is an example [205]. The hierarchy of models of the action potential reach from the atomic origin of its voltage sensor, through the channels that control current, to the current flow itself and how it produces a signal that propagates meters.

Biology requires analysis from atom size to arm length [132,206,207,208,209,210,211,212,213,214]. A general analysis from Ångstroms to meters is made possible by structures at every scale. The enormous range and density of structures in biology creates a hierarchy in which analysis is possible [134]. Analysis that follows the path of those structures is following the path of natural selection. Analysis that follows the path of those structures—like the living beings it analyzes—can survive, succeed, and reproduce where general analysis is inconceivable.

It is vital to realize that these general words lead to specific analysis of experimental systems of considerable complexity and importance in health and disease. Precise analysis of experiments and prediction of yet unmeasured results are possible with few adjustable parameters. The large number of parameters in the equations are often known from biophysical, stereological and other anatomical measurements.

These complex systems include ion channels, cells and tissues as complex as skeletal muscle or the lens of the eye and even systems involving many cell types like the optic nerve bundle of the central nervous system. The systems include the enormously important proteins that generate ATP in mitochondria, e.g., cytochrome c oxidase.

The central role of structure is evident in this analysis whether on the scale of individual protein channels, transporters, or on the scale of a bundle of nerve fibers. That structure must be present in a theory if the special role of structure is to be exploited. Theories embed structures in the structures of their boundaries, and the physical laws followed at those boundaries. Theories embed structures in the shape and properties of their boundary conditions. Electrodynamics requires boundary conditions so it easily accommodates the role of structure in biology. Classical statistical mechanics does not, I am sorry to say. A statistical mechanics extended to involve structures and their boundary conditions can deal with the constraints of structure and thus make analysis much easier.

**Setting boundaries**. The boundaries I propose for statistical mechanics are easy to enumerate

1. The boundary treatments must be compatible with electrodynamics because the equations of electrodynamics are universal and exact when written in the form of the Core Maxwell Equations Equations (2)–(5).
2. Structures and boundaries must be involved, that describe the system and specific experimental setup used for measurement, albeit in an approximate way.
3. Systems with known function, of known structure, should be studied first. These often dramatically simplify problems, as they were designed to do, by engineers or evolution, once we known how to describe and exploit the simplifications using mathematics.
4. Systems that are devices, with well defined inputs, outputs, and input-output relations, should be identified because their properties are so much easier to deal with than systems and machines in general. Fortunately, devices are found throughout living systems, albeit not always as universally (or as clearly defined) as in engineering systems [132,133,134].

When statistical mechanics is used without bounds, it is a quicksand which cannot support a hierarchy of models. Statistical mechanics without bounds is a dangerous foundation for structures with charge. They are likely to fail because the fields produced by charges depend on boundaries and the conditions at those boundaries.

When statistical mechanics is used within bounds, the quicksand is constrained within retaining walls, and the foundation and structures of our models can become strong and useful. Retaining walls provide strong foundations even for skyscrapers. Retaining walls make civilization possible in lands below sea level.

Statistical mechanics within bounds can provide the foundation so badly needed for our models of biological and biochemical systems.

Electrodynamics is always a safe foundation. Statistical mechanics can take its rightful place alongside electrodynamics once it is bound within structures and the conditions at those boundaries.

**One way to set boundaries in statistical physics.** One way to set boundaries for statistical mechanics is to fulfill the dream of Katchalsky and Curran [215] shared with a Harvard undergraduate in 1962 (Bob Eisenberg, personal communications). They hoped to see a full fledged field theory that would replace classical statistical mechanics, and allow flows driven through dissipative systems by many forces, electrical, diffusional and convective, even thermal. Such a field theory would include boundary conditions as an inescapable component although the importance of such conditions was not mentioned by Katchalsky, as far as I know. Their basic plan was to build on the work of John Strutt (with later alias Lord Rayleigh) that analyzed purely dissipative systems (without conservative forces (Strutt did not have a well developed field theory built on partial differential equations and variational calculus to use. Rather, he had to use ordinary differential equations, at least that is my view of the history)) [216,217,218] as Onsager [219,220,221,222] had attempted. Onsager attempted to include conservative forces in that treatment.

The mathematical issues were formidable [137,143,164,223,224,225,226,227,228,229], and required easy combination of variations of conservative forces (hopefully in Eulerian coordinates because conservative forces are often functions of position most naturally) and variations of dissipative forces (hopefully in Lagrangian coordinates, because friction is a function of velocity and flow). Onsager did not have a well developed variational calculus to build on that included pull back and push forward techniques to switch between Eulerian (fixed in space) and Lagrangian (moving) coordinates. The variational calculus, and partial differential equations of field theory, became routine tools of applied mathematics much later [155], in the MIT curriculum [154], for example.

A fully consistent variational treatment including conservative and dissipative forces has been developed by Chun Liu, more than anyone else, and a tutorial introduction [230] and reviews (e.g., [56,231]) are available for those who wish to try this approach. The application of EnVarA to ionic systems—that were of such interest to Katchalsky and Curran—were focused on ion channels in [54], where the name EnVarA (Energy Variational Approach) was introduced, following earlier work [143,232,233] reviewed and expanded in [53,55,234] and elsewhere [56,230,231].

When applied to ionic solutions, particularly in the context of ion channels, the EnVarA approach is a successful beginning, combining statistical mechanics and electrostatics. It has been extended to include chemical reactions as described in the traditional rate constant formulation [55,199,231,235]. However, references [236,237] show the need to eventually include the dependence of rate constants on the electric field.

An extension of chemical kinetics to include the quantum mechanical origin of rate constants would of course be most valuable. However, quantum chemistry in ionic solutions is not available in the EnVarA formulation or anywhere else, as far as I know. It does not seem feasible quite yet because an extension to the quantum chemical domain must include electrodynamics even in the far field if it is to deal with chemical reactions in ionic solutions. The number of atoms involved then is far beyond what can be integrated in numerical treatments of quantum mechanics, whether the Schrödinger equation, density functional, or hybrid experimental simulation approaches, as far as I know.

Challenges remain in extending the EnVarA approach to other systems of practical interest. EnVarA needs to deal with the time dependent problems of electrodynamics (and the Maxwell equations) if it is deal with molecular dynamics of proteins. Proteins, like genes, have functions controlled by a handful of atoms. Atomic scale analysis and simulations are thus required to understand proteins, genes, and the great majority of biological functions that are directly controlled by proteins.

Atomic motions are simulated in many laboratories interested in how proteins work. These simulations customarily pretend that electrostatics is sufficient to calculate electrodynamics occurring on the femtosecond time scale. They almost always use Coulomb’s law, despite the rapid motion of atoms. As Feynman says (Section 15-6 of volume 2 of [4]) in vivid language, over some five pages (Figure 1), Coulomb’s law is false when charges are moving rapidly. Coulomb’s law is valid only in electrostatics. Femtosecond time scales are clearly not electrostatics. The errors in assuming Coulomb’s law for atomic motions cannot be expected to be negligible although they may not be important in some circumstances.

Like Coulomb’s law EnVarA at present is confined to the electrostatic field. Thus this field theory approach to statistical mechanics and chemical reactions [55,199,231,235] is also confined to electrostatics. This is reasonably successful for ionic solutions because it allows boundaries and boundary conditions even if only of an electrostatic type. Note that membrane capacitance can be included as it must in any reasonably adequate treatment of bioelectricity [156,238,239,240,241]. Treatments of apparently stationary problems (in dealing with convection for example) need to involve membrane capacitance [242], although this has not always been done, following [243]. We need to learn to use the full Maxwell equations in our variational treatment to deal with molecular dynamics and motions of the handful of atoms that control specific functions of proteins.

Nonideal properties of ionic solutions have also not been included in an EnVarA treatment in a general form, despite their evident importance in all the ionic solutions of life (blood, plasma, extracellular and intracellular fluids that cells, organs, and tissues live in). Two specific formulations are in [54] but a general treatment is not yet complete that deals with differential capacitance, variations of ionic activity with the concentration and composition of ionic solutions, and the conductance of mixed solutions of various concentration and composition. The nonideal effects arise mostly from the finite diameter of ions (and their shape in general) which change the electric field dramatically compared to that in ideal solutions of ions. The reviews of [244,245] are gateways to the huge literature of nonideal properties, highlighted for biophysicists in [246,247].

As one could imagine, effects of finite size are particularly important near the interfaces, that form the boundaries of ionic solutions and the electrodes that supply current and measure potential. Protein binding sites and the active sites of protein enzymes have enormous surface to volume ratios [183] and depend on electrostatics to control their function [248,249,250,251] as vividly shown by the work of Boxer and his collaborators [252,253,254,255,256].

Ion channels are all interfaces—if one can be forgiven some vivid language—so the effect of finite size and crowding of ions in channels has become a central issue [257,258] since it was first introduced [184,247,259] and used with Monte Carlo techniques [185,260] to explain and design the selectivity of calcium and sodium channels [261,262,263,264,265]. Recent field theories include several treatments of finite size [54]. Other treatments of finite size have not yet been extended into a field theory formulation although [186,266] point the way.

Ions are crowded where they are most important [184,259] in many systems beyond ion channels and so these issues have attracted much attention in the literature of nanosystems, artificial channels, super capacitors, and so on. Reviews of Jinn-Liang Liu provide a gateway to the immense literature on finite volume effects [186,267] but work is so active [268,269,270], and the literature is expanding so rapidly, that readers must depend on their own searches of the literature. Finite size effects are important in more or less any application involving ions.

## 5. Conclusions

It seems then that bounds can be included in classical statistical mechanics if the traditional approaches starting with equilibrium distribution functions are replaced with the appropriate field theories, including the multi-physics of diffusion, convection, heat conduction, and migration, built on the bedrock of the Maxwell equations of electrodynamics. Field theories automatically include boundary conditions.

Important issues remain: atoms move quickly and analysis must be extended to deal with electrodynamics, not electrostatics.

## Data Availability

Not applicable.
